# Enzymatic Antioxidant System Activation Assures the Viability of *Guadua chacoensis* (Bambusoideae, Poaceae) Embryogenic Cultures during Cryopreservation

**DOI:** 10.3390/plants12030673

**Published:** 2023-02-03

**Authors:** Luiza Giacomolli Polesi, Daniela Goeten, Hugo Pacheco de Freitas Fraga, Neusa Steiner, Miguel Pedro Guerra

**Affiliations:** 1 Graduate Program in Plant Genetic Resources, Laboratory of Plant Developmental Physiology and Genetics, Federal University of Santa Catarina, Florianópolis 88034-001, SC, Brazil; 2 Departament of Botany, Federal University of Santa Catarina, Florianópolis 88040-535, SC, Brazil; 3 Graduate Program in Botany, Federal University of Paraná, Curitiba 81531-980, PR, Brazil; 4 Graduate Program in Agricultural and Natural Ecosystems, Federal University of Santa Catarina, Curitibanos Campus, Ulysses Gaboardi Road, Km 3, Curitibanos 89520-000, SC, Brazil

**Keywords:** bamboo, in vitro conservation, somatic embryogenesis, suspension cultures

## Abstract

This study aimed to establish a cryopreservation protocol for *G. chacoensis* embryogenic cultures (ECs) and to investigate the role of antioxidant enzymes activities during cryopreservation. The growth dynamics of cell suspensions were also investigated, followed by a phytotoxicity test to assess the ECs’ ability to tolerate the use of cryoprotective solutions for different incubation times (0, 30, 60, 120, and 240 min). We evaluated the EC redox state in three steps of cryopreservation: after incubation in cryoprotection solution, after thawing, and 60 days after regrowth. Our results showed that the ECs support the use of cryoprotective solution until 120 min, showing phytotoxic effects with 240 min of incubation. This study reports a 100% survival of the cultures and a 10% increase ratio in fresh material for both incubation times tested (60 and 120 min). Increased malonaldehyde content was identified after incubation in the cryoprotective solution. An increase in the activities of catalase and ascorbate peroxidase was also identified in the subsequent steps, suggesting that the activation of antioxidant enzymes is essential for maintaining cell homeostasis during cryopreservation.

## 1. Introduction

Cryopreservation is defined as the conservation of germplasm in ultra-low temperatures (−196 °C), normally in liquid nitrogen (LN), thus allowing the preservation of material using a small space while presenting a low contamination risk and requiring minimal maintenance [1,2]. In addition, cryopreservation permits the conservation of material for long periods with the assurance of genetic stability since it is believed that all metabolic and physiological processes are arrested under these conditions [1,2,3,4].

Cryopreservation is emerging as a useful strategy for the long-term and safe conservation of embryogenic cultures (ECs) generated in somatic embryogenesis protocols, as the maintenance of these materials under cryopreserved conditions preserves cell viability without the loss of embryogenic potential [5]. Indeed, this strategy has been constantly applied for the cryopreservation of somatic embryos of different plant species [6,7,8,9]. For bamboo, an important group of the grass family, somatic embryogenesis represents a suitable strategy of in vitro propagation as it solves difficulties faced by conventional propagation methods, such as damage to the mother plant and irregular or not always viable seed production [10]. The association of this technique with cryopreservation represents a perfect match for bamboo propagation and conservation; however, no reports exploiting cryopreservation and somatic embryogenesis have been described [11]. Only two reports on the slow-growth conservation of somatic embryos of Dendrocalamus hamiltonii [12,13] and one on cryopreservation of the whole zygotic seed [14] were available to date, demonstrating the need for new strategies for the in vitro conservation of bamboo species.

*Guadua chacoensis*, the species studied in this work, is a lignified bamboo (*Bambusoideae*) presenting natural distribution in Argentina, Bolivia, Brazil, and Paraguay [15]. Its seed production occurs irregularly and in long cycles of approximately 28 years [16]. The use of in vitro biotechnology tools such as somatic embryogenesis and cryopreservation are fundamental for its propagation and conservation.

Over more than 60 years of using cryopreservation, various methodologies have been established, i.e., classical slow freezing, encapsulation/ dehydration, vitrification, droplet vitrification, and D and V cryo-plate methods [2]. The classical slow-freezing method, also known as the two-step freezing technique, includes pre-growth with osmotic agents to acclimate cells and increase freezing tolerance, and the cryoprotective treatment. Additionally, during the freezing step, the use of a Mr. Frosty unit allows for the slow cooling of the material, providing a decrease of 1 °C per min until a temperature of −40 °C is achieved. This is followed by direct immersion in liquid nitrogen [5,17,18]. Overall, the primary purpose of cryopreservation, independent of the methodology applied, is to control dehydration in order to avoid cryoinjuries during the freezing and thawing steps [18].

Low temperatures during the cryopreservation process can impose stress on plant cells, including physical damage, changes in membrane viscosity, retardation of metabolic activity, and the formation of radicals that lead to oxidative stress [19]. Among the physical damages, the most critical effect is the disruption of membranes and disconnection of the cells as a consequence of intracellular ice crystal formation [2]. One way to prevent this is by using cryoprotective agents (CPAs) that can permeate cell membranes, including DMSO and glycerol, and non-permeable agents, including polyethylene glycol and sucrose [5]. The exact method by which CPAs act in the cell is still unclear. However, it is suggested that CPAs, particularly the permeable ones, are able to increase cell osmolarity and reduce cell water content, resulting in better cell tolerance to dehydration [17]. Moreover, the use of these agents may facilitate the transition of the water phase directly to the vitrification phase, also known as the glassy state, without the formation of ice crystals [2].

The generation of reactive oxygen species (ROSs), such as hydrogen peroxide (H_2_O_2_), superoxide radical (O_2_^•−^), hydroxyl radical (OH^•^), and singlet oxygen (^1^O_2_) [20], is inevitable during the multiple steps of cryopreservation [21,22]. An excessive imbalance between ROS production and the activity of antioxidant enzymes, such as superoxide dismutase (SOD), catalase (CAT), ascorbate peroxidase (APX), and guaiacol peroxidase (GPX), could result in oxidative stress, which disrupts cellular homeostasis and can lead to membrane damage by lipid peroxidation, protein oxidation, DNA damage, and, in the most serious case, results in programmed cell death [22]. In addition, high oxidative stress can negatively affect cryopreservation and might lead to reduced culture viability and recovery rates [9,23,24,25].

In our previous study, we described and characterized the somatic embryogenesis of *Guadua chacoensis* using morphohistological and biochemical features [26]. Therefore, the present study aims to establish a cryopreservation protocol for *G. chacoensis* ECs using the classical two-step freezing procedure and to characterize the role of antioxidant enzymes in the cryopreservation process.

## 2. Results

### 2.1. Growth Dynamics Revealed A Long Growth Phase for the G. chacoensis EC

**Figure 1:**
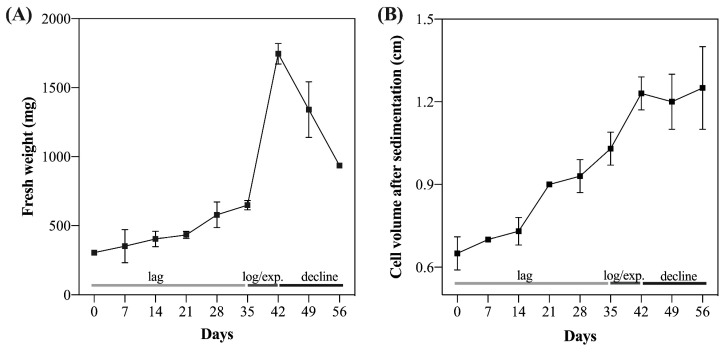
Growth dynamics of *G. chacoensis* embryogenic cultures measured by a destructive method (**A**) and non-destructive method (**B**). (**A**) Growth determined by fresh weight measurement (mg mg^−1^) and (**B**) growth determined by cell volume after sedimentation (CVS) (cm). Bars indicate the standard deviation of the mean. Lag: lag phase; log/exp: logarithmic or exponential phase; decline: decline phase.

An analysis of the growth dynamics using both destructive (Figure 1A) and non-destructive (Figure 1B) methods revealed that the *G. chacoensis* ECs were in the lag growth phase between days 0 and 35 of culture, followed by the exponential or logarithmic phase from days 35 to 42 and the decline phase after day 42. Our results also showed that the increased ratio of fresh material during the exponential phase achieved almost six times the initial weight (Supplementary Figure S1A), while the increment in the cell volume after sedimentation was almost two times higher (Supplementary Figure S1B).

### 2.2. G. chacoensis EC Support 120 Min Incubation in the Cryoprotective Solution

**Figure 2:**
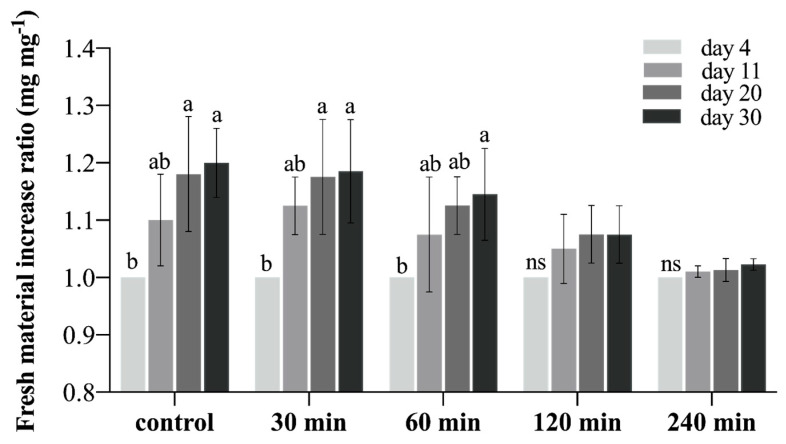
Fresh mass increased ratio (mg mg^−1^) of *G. chacoensis* embryogenic cultures subjected to different incubation times (control–0, 30, 60, 120, and 240 min) in cryoprotective solution after 4, 11, 20, and 30 days. Bars indicate the standard deviation of the mean. Lowercase letters represent significant differences between dates in each incubation time, according to the Tukey test (*p* < 0.05). ns: not significant.

**Figure 3:**
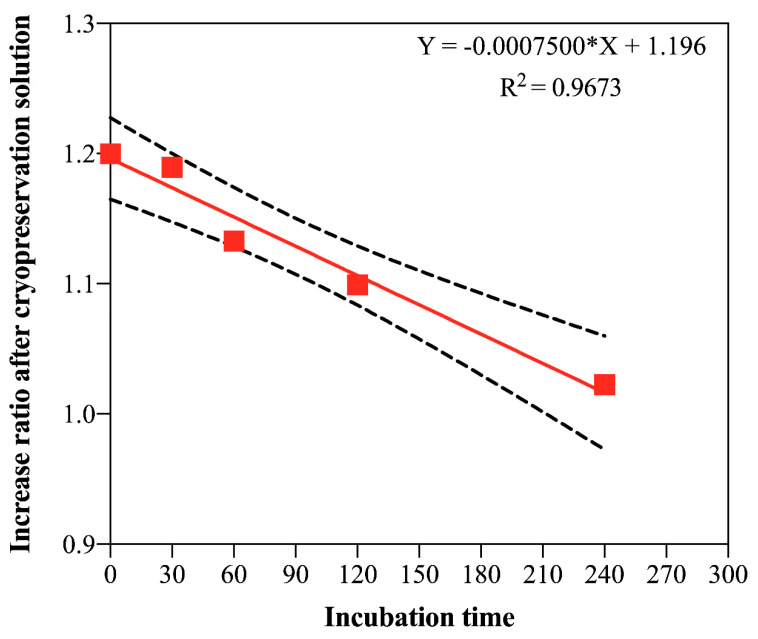
Linear regression showing the increased ratio in fresh material of *G. chacoensis* embryogenic cultures 30 days after incubation (0, 30, 60, 120, and 240 min) in cryoprotective solution. * Indicates that linear regression was significant (F-test, *p* ≤ 0.05).

The results of the phytotoxicity evaluation revealed that the ECs incubated for 30 and 60 min in the cryoprotective solution presented a fresh material increase ratio similar to the control (not subjected to cryoprotective solution) along all the evaluation times (Figure 2). The same behavior could also be observed on day 30 in the linear regression analysis (Figure 3), suggesting that these incubation times do not affect the cultures’ growth dynamics. In the same way, the incubation period of 120 min presented similar results to the control until day 20; however, on day 30, the fresh material increase ratio was significantly lower when compared to the control (Figure 2 and Figure 3). Inversely, the incubation period of 240 min was considered phytotoxic for the *G. chacoensis* ECs, as the growth was practically null along the evaluation time (Figure 2 and Figure 3). Based on these results, we chose the incubation times of 60 and 120 min to be used in the complete cryopreservation procedure.

### 2.3. Regrowth Rates and FDA Staining Indicate the Establishment of A Highly Efficient Protocol for Cryopreservation of G. chacoensis ECs

**Figure 4:**
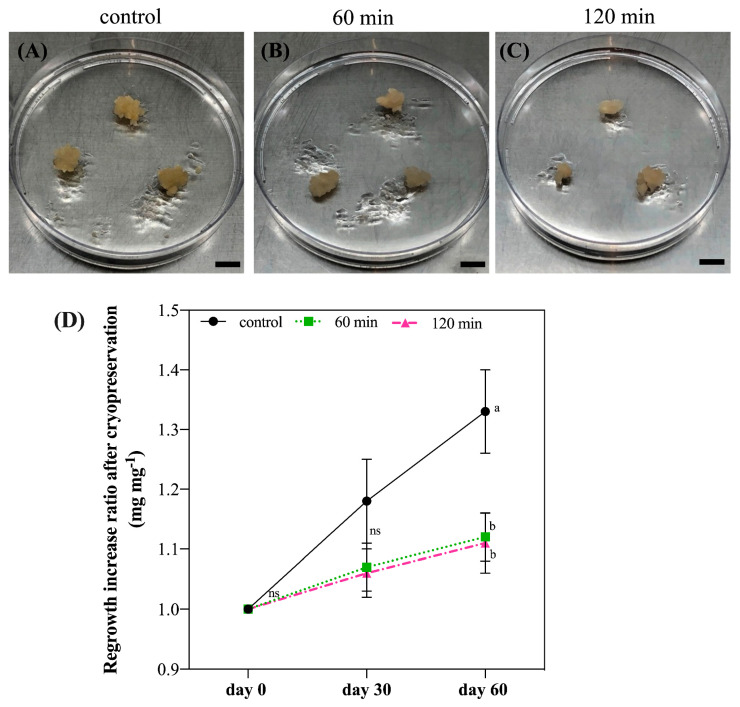
Regrowth of *G. chacoensis* embryogenic cultures after cryopreservation procedure: (**A**) control after 60 days; (**B**) 60 min incubation culture at 60 days: (**C**) 120 min incubation culture at 60 days; (**D**) regrowth increase ratio (mg mg^−1^) at 30 and 60 days after cryopreservation for control, 60 min and 120 min incubation cultures. Bars indicate the standard deviation of the mean. Lowercase letters represent significant differences between incubation times on each day, according to the Tukey test (*p* < 0.05). ns: not significant. Bar (**A**–**C**): 1 cm.

The analysis of the cultures’ regrowth 60 days after cryopreservation (Figure 4A–C) revealed a 100% regrowth and a 10% increase in fresh material for both incubation times tested—60 and 120 min (Figure 4D)—demonstrating success in the use of this protocol for cryopreservation of *G. chacoensis* ECs.

**Figure 5:**
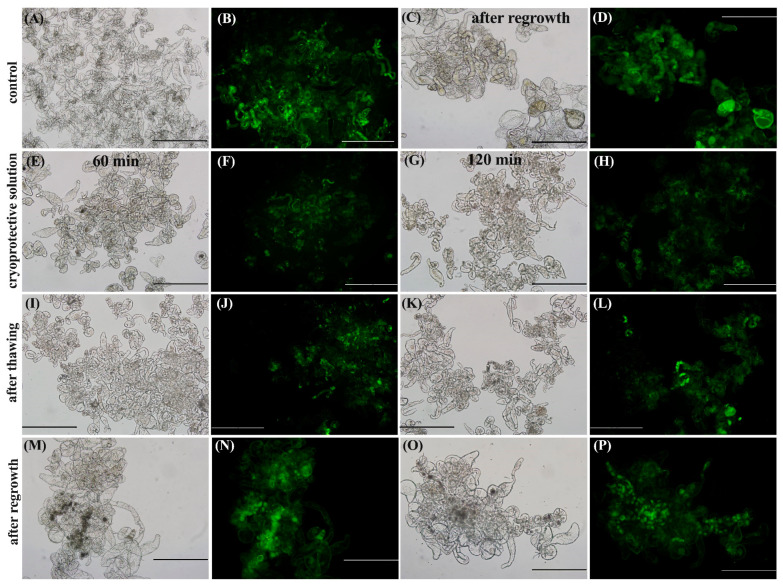
FDA staining of *G. chacoensis* embryogenic cultures showing the cell viability of the control (**A**,**B**), control after regrowth (**C**,**D**), after the use of cryoprotective solution (**E**–**H**), after thawing (**I**–**L**), and after 60 days of regrowth (**M**–**P**), for 60 min incubation (**E**,**F**,**I**,**J**,**M**,**N**), and 120 min incubation (**G**,**H**,**K**,**L**,**O**,**P**). Letters A, C, E, G, I, K, M, and O show the bright field, while letters B, D, F, H, J, L, N, and P show the fluorescein diacetate. Bars: 500 μm.

An FDA analysis of the control treatment (Figure 5A,B) showed several regions with high fluorescence emissions. For the cryoprotective solution, the images revealed some embryogenic cells together with some vacuolated cells in a bright field of 60 min (Figure 5E) and 120 min (Figure 5G). Only the embryogenic cells exhibited a clear FDA fluorescence signal (Figure 5F,H), suggesting that the use of a high-osmolarity cryoprotective solution may be causing osmotic stress that leads to the loss of some cell integrity, especially in the 120 min incubation period (Figure 5H).

In the analysis of the following step, after thawing (in which the culture had already been subjected to slow cooling in Mr. Frosty, freezing in LN, and thawing), the presence of clusters of embryogenic cells presenting fluorescence increased, especially for the 60 min incubation period (Figure 5I,J), in relation to the cryoprotective step. Regarding the 120 min incubation period, it seems some cells demonstrated cell division and FDA staining, indicating that they maintained cell viability (Figure 5K,L).

At 60 days after regrowth, the imagens exhibited the presence of clusters of embryogenic cells in a bright field, indicated in Figure 5M,O, and the presence of strong FDA staining in these same clusters (Figure 5N,P), independent of the incubation time used. Additionally, this behavior is the same observed for the control treatment after regrowth (Figure 5C,D).

Our results suggested that, although the *G. chacoensis* ECs may suffer some stress during their incubation in the cryoprotective solution, the viability of the cells was maintained. Additionally, the highest viability was observed 60 days after regrowth, suggesting a successful establishment of the cryopreservation procedure for this culture.

### 2.4. Analysis of the Content of MDA, H_2_O_2_, and the Activity of Antioxidant Enzymes Revealed the Importance of the Enzymes CAT and APX during Cryopreservation

**Figure 6:**
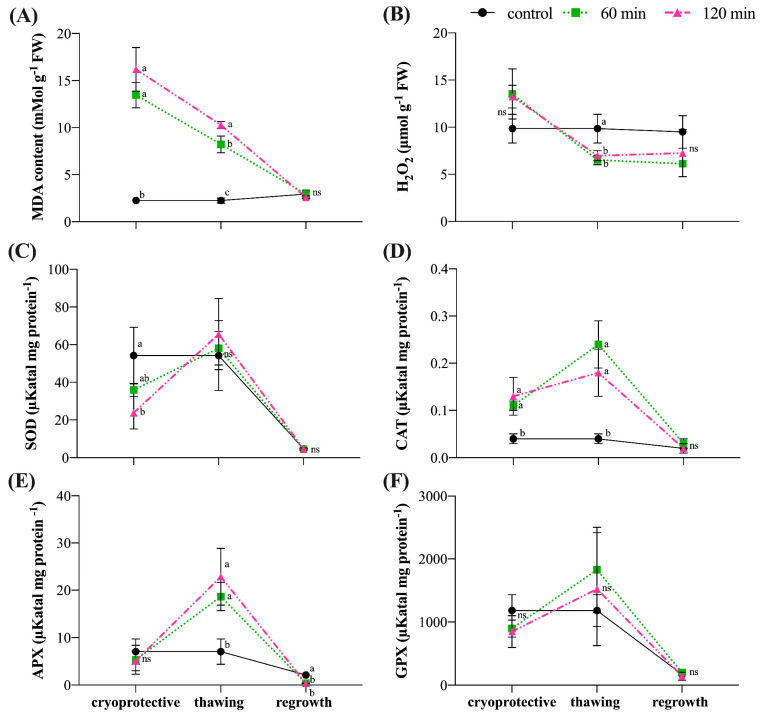
Redox states of *G. chacoensis* embryogenic cultures, characterizing the steps of the cryoprotective solution, thawing, and 60 days’ regrowth for the control, 60 min incubation, and 120 min incubation: (**A**) malonaldehyde (MDA) content; (**B**) hydrogen peroxide (H_2_O_2_) content; (**C**) superoxide dismutase (SOD) activity; (**D**) catalase (CAT) activity; (**E**) ascorbate peroxidase (APX) activity; and (**F**) guaiacol peroxidase (GPX) activity. Bars indicate the standard deviation of the mean. Lowercase letters represent significant differences between incubation times on each step, according to the Tukey test (*p* < 0.05). ns: not significant.

The incubation of the *G. chacoensis* ECs in the cryoprotective solution resulted in an MDA content 6- and 7-fold higher than the control for the 60 and 120 min incubation periods, respectively (Figure 6A). Although these values decreased by approximately 40% in the thawing step, they are still significantly higher than the control. At this time, 120 min of incubation presents the highest MDA content (Figure 6A). Our results also showed that, at 60 days after regrowth, no significant difference was seen in MDA content from both incubation times and the control (Figure 6A).

The H_2_O_2_ contents presented no significant difference among the treatments in the cryoprotective treatment steps (Figure 6B). However, the values for the 60 min and 120 min were significantly lower than for the control in the thawing steps (Figure 6B). At regrowth, no differences were seen between the H_2_O_2_ contents (Figure 6B).

Quantification of SOD showed a significant difference only in the cryoprotective treatment step, whereas SOD activity was lower for the 120 min incubation experiment in relation to the control (Figure 6C).

CAT activity was 3-fold higher following cryoprotective treatment for 60 min and 120 min in relation to the control. These values increased in the thawing steps, becoming 6.5- and 4.5-fold higher in 60 and 120 min, respectively, in relation to the control (Figure 6D). However, no significant difference was observed at the regrowth stage (Figure 6D).

APX activity did not present a significant difference in the cryoprotective and regrowth steps. Conversely, at the freezing step, APX activity was approximately 3-fold higher for the 60 min and 120 min incubations when compared to the control (Figure 6E).

GPX activity did not present a significant difference between the treatments and different steps of the cryopreservation protocol (Figure 6F).

A correlation analysis revealed a strong positive correlation between MDA and CAT (Pearson’s R: 0.906, *p*: 0.001) in the incubation of the cultures in cryoprotective solution (Table 1), suggesting that an increase in MDA content leads to an increase in CAT content and vice versa (Figure 6A,D). The same behavior was also seen for the MDA and APX content (R: 0.724, *p*: 0.027) (Figure 6A,E) and the CAT and APX content (R: 0.821, *p*: 0.007) (Figure 6D,E) at the thawing step (Table 1). On the contrary, a strong negative correlation was observed between MDA and H_2_O_2_ (R: −0.797, *p*: 0,010) (Figure 6A,B) at this same step, suggesting that as the MDA content decreases, the H_2_O_2_ content also decreases. Additionally, a negative and inverse correlation was also observed for the CAT and H_2_O_2_ content (R: −0.770; *p*: 0.015) at thawing steps (Table 1), in which an increase in the CAT content resulted in a decrease in the H_2_O_2_ content (Figure 6B,D). No strong correlation was observed between the data at the regrowth step (Table 1).

## 3. Material and Methods

### 3.1. Plant Material

Embryogenic cultures (ECs) of *G. chacoensis*, obtained as previously described by Giacomolli Polesi et al. [26], were used as plant material for the establishment of suspension cultures. To establish the suspension cultures, 300 mg of ECs were inoculated in 250 mL Erlenmeyer flasks containing 50 mL of suspension culture medium (SCM), which was composed of MS basal salts [27] supplemented with L-glutamine (1 g L^−1^), sucrose (30 g L^−1^), Morel vitamins (2 mL L^−1^) [28], and Picloram (10 μM). The pH of the culture medium was adjusted to 5.8 before autoclaving at 121 °C and 1.5 atm for 15 min. The Erlenmeyer flasks were kept in dark conditions, using an orbital shaker to achieve permanent agitation (95 rpm), in the growth room at 25 ± 2 °C for 45 days.

### 3.2. Analysis of Cell Growth Dynamics of Suspension Cultures

To characterize the growth phases of *G. chacoensis* ECs (lag, exponential, or logarithmic, linear, and decline), two different parameters were analyzed: a destructive method, measuring the ECs’ fresh weight; and a non-destructive method, measuring the cell volume after sedimentation using a device developed for suspension-culture sedimentation with a ruler, proposed by Mustafa et al. [18]. Thus, 24 replicates of a 250 mL Erlenmeyer flask containing 300 mg of *G. chacoensis* EC and 50 mL of SCM were used. Both measures were performed every 7 days until day 56 of culture, when the growth ratio was obtained using the following equations: (i) fresh weight at day x/the initial fresh weight; and (ii) cell volume after sedimentation at day x/initial cell volume after sedimentation.

### 3.3. Phytotoxicity Evaluation of Cryoprotective Solution

Following the methodology proposed by Mustafa et al., a phytotoxicity test was utilized to evaluate the possible toxicity of a cryoprotective solution for *G. chacoensis* ECs [18]. The cryoprotective solution was composed of 2 M of sucrose, 1 M of glycerol, 1 M of dimethylsulfoxide (DMSO), and 1% proline (weight: volume). The final two components were filter esterized in a flow chamber in the cool autoclaved solution (121 °C, 1.5 atm for 12 min). In this bioassay, ECs were subjected to five incubation times (0, 30, 20, 120, and 240 min) without being subjecting to the freezing step, as was proposed by Mustafa et al. [18] and Fraga et al. [7], with modifications. Four replications of the Erlenmeyer flasks, each containing 1 g of *G. chacoensis* ECs and 50 mL of SCM at day 39 of subculture, were used for each incubation time, totaling 20 Erlenmeyer flasks.

Briefly, 25 mL of SCM was removed and replaced by the same volume of pretreatment solution, which consisted of the basal SCM medium (Picloram-free) plus mannitol (180 g L^−1^). The suspension cultures were pre-incubated for two days in an orbital shaker at 95 rpm and 25 ± 2 °C in dark conditions. The cultures were then kept in the device until the sedimentation of the cells and the removal of the culture medium above the cells (approximately 25 mL). A cold, cryoprotective solution (0 °C) was added in the same volume that was removed, and the five incubation times were tested.

The incubation process was performed in an orbital shaker at 95 rpm and 0 °C. We then used a sterilized funnel, attached to a Kitasato flask with a vacuum pump, for the suction of the liquid medium and collection of the *G. chacoensis* ECs. These cultures were transferred to Petri dishes containing two filter-paper disks and 25 mL of MSC medium gelled with agarose (7.5 g L^−1^). After 2 days of incubation, the upper filter paper with the EC was subcultured to a fresh SCM medium gelled with agarose (7.5 g L^−1^), and. After 7 days, the cultures were transferred to SCM gelled with phytagel (2 g L^−1^) without filter paper. The cultures were kept in constant darkness at 22 ± 2° C.

The *G. chacoensis* ECs were weighted at 4, 11, 20, and 30 days of culture, and the final weight-increased ratio after the phytotoxicity test was obtained using the following equation: fresh weight at day 30/fresh weight at day 4.

### 3.4. Cryopreservation Experiments

**Figure 7:**
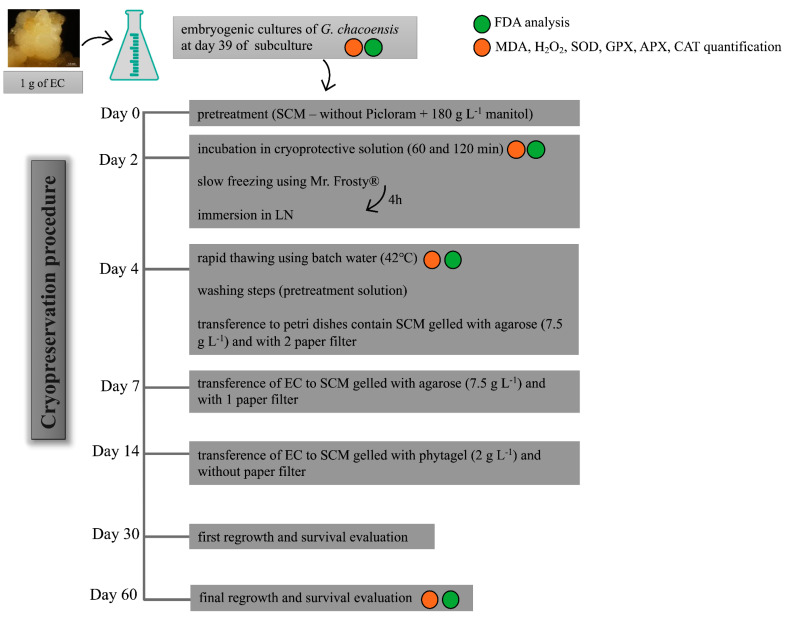
Cryopreservation procedure used for embryogenic cultures of *G. chacoensis,* described step-by-step. EC: embryogenic culture; SCM: suspension culture medium; FDA: Fluorescein diacetate; MDA: malonaldehyde; H_2_O_2_: hydrogen peroxide; SOD: superoxide dismutase; CAT: catalase; APX: ascorbate peroxidase; and GPX: guaiacol peroxidase. Circles in green and orange identify the steps during which FDA analysis and enzymes analysis were realized, respectively.

After evaluating the toxicity of the cryoprotective solution and defining the best incubation times, the complete cryopreservation protocol was performed according to Mustafa et al. [18] and Fraga et al. [7], with adaptations. The step-by-step cryopreservation procedure used in the present study is shown in Figure 7.

*G. chacoensis* ECs, at 39 days of culture and in the exponential phase, were used as plant material. The experiment was realized using five replicates for each incubation time, totaling 15 Erlenmeyer flasks. The initial steps of the protocol were the same as described above, whereas the suspension cultures were first incubated for 2 days in a pretreatment solution in an orbital shaker at 95 rpm and 25 ± 2 °C in dark conditions. This was followed by incubation in the cryoprotective solution for 60 and 120 min in an orbital shaker at 95 rpm and 0 °C. After this period, the *G. chacoensis* ECs were collected in a sterilized funnel with a filter paper attached to a Kitasato flask, using a vacuum pump for the suction of the liquid medium and cryoprotective solution. Next, approximately 300 mg of the collected ECs was transferred to each cryovial (2 mL), totaling 18 cryovials from each incubation time. These were transferred to Mr. Frosty (Nalgene^®,^ Waltham, MA, USA) containers containing 250 mL of isopropanol. The containers were maintained in an ultra-freezer (−80 °C) for 4 h for controlled cooling according to the manufacturer’s instructions. Afterward, the cryovials were immediately transferred to liquid nitrogen and were kept immersed for at least 24 h.

The subsequent step involved the rapid thawing of the cultures through the immersion of the cryovials in a deionized water bath at 40 °C for approximately 2 min. The cryotubes were kept on ice for a few minutes until the cultures decanted to the bottom of the tubes. Thus, the superior phase without cultures was removed and replaced by the same volume (1 mL) of washing solution (pretreatment solution—SCM plus mannitol (180 g L^−1^)). Next, the cultures were decanted again, the washing solution was discarded, and the cultures were transferred to Petri dishes containing two filter-paper disks and 25 mL of SCM medium gelled with agarose (7.5 g L^−1^). After 3 days of incubation, the upper filter paper with the *G. chacoensis* cultures was subcultured to a fresh SCM medium gelled with agarose. Subsequently, after 7 days, the cultures were transferred to SCM gelled with phytagel (2 g L^−1^) without filter paper. The cultures were kept in constant darkness at 22 ± 2 °C for 60 days.

The EC regrowth was evaluated at 30 and 60 days after the cryopreservation procedure for each incubation time. A control treatment, which consisted of a cell suspension of *G. chacoensis* ECs that were not subjected to the cryopreservation protocol, was used as a control for the regrowth analysis. The experiment was carried out in a completely randomized design, with six Petri dishes containing three colonies of *G. chacoensis* ECs each, and the regrowth rate was evaluated using the following equation: fresh weight at day 30 or 60/initial fresh weight.

For the quantification of the MDA and H_2_O_2_ content and the determination of antioxidant enzyme activity (the activity of SOD, CAT, APX, and GPX), samples were collected in three different stages of the cryopreservation protocol: (i) after incubation in cryoprotection solution; (ii) after freezing and thawing; and (iii) 60 days after regrowth (Figure 7). Controls corresponding to the suspension culture that were not subjected to the cryopreservation procedure for steps (i) and (ii) and this same material were collected, transferred to Petri dishes on day 4, and then passed through all the following steps until day 60 regrowth (Figure 7), corresponding to the control of step (iii).

### 3.5. Quantification of MDA and H_2_O_2_ Content

The quantification of the MDA and H_2_O_2_ content was performed according to Velikova et al. [29], with an adaptation described by Polesi et al. [30].

Briefly, three samples of 200 mg of fresh material were collected from each incubation time (60 or 120 min) and the control treatment. The extraction procedure was performed through the maceration of the samples, followed by homogenization with 0.1% trichloroacetic acid (TCA) (1/5; *w*/*v*) and centrifugation at 12,000 rpm and 4 °C for 20 min. The supernatant collected was used as a culture substrate in the reaction mixture for the determination of the MDA and H_2_O_2_ content.

Contents of MDA were quantified using the thiobarbituric acid (TBA) method, in which 200 μL of the culture extract was mixed with 400 μL of a buffer containing 20% (*w*/*v*) trichloroacetic acid (TCA) and 0.5% (*w*/*v*) TBA and incubated in bath water at 95 °C for 30 min. After that, the microtubes were placed on ice for 15 min, followed by centrifugation at 10,000 rpm for 15 min at 4 °C and the subsequent collection of the supernatants. The MDA concentration was determined by reading 200 μL of the mixture in a spectrophotometer (SpectraMax Paradigm, Molecular Devices, San Jose, CA, USA) at 532 nm, adjusted for nonspecific absorbance at 600 nm using an extinction coefficient of 155 mM cm^−1^. The contents were expressed in μmol g^−1^ of fresh material.

For the quantification of H_2_O_2_ contents, 200 μL of the culture extract was mixed with 100 μL of 10 mM potassium phosphate buffer (pH 7.0) and 200 μL of 1 M potassium iodide and kept for 15 min at room temperature for the activation of the reaction. The H_2_O_2_ content was then determined by reading 200 μL of the mixture in a spectrophotometer (SpectraMax Paradigm, Molecular Devices, San Jose, CA, USA) at 350 nm, and the concentration was assessed using a known, standard curve of H_2_O_2_. The contents were expressed in μmol g^−1^ of fresh material.

Using GraphPad Version 8.1.1, data obtained for the MDA and H_2_O_2_ content were submitted to a one-way analysis of variance (ANOVA) and the Tukey mean separation test (*p* < 0.05).

### 3.6. Antioxidant Enzymes Activity

The determination of the activity of antioxidant enzymes superoxide dismutase (SOD), catalase (CAT), ascorbate peroxidase (APX), and guaiacol peroxidase (GPX) was realized following the methodology proposed by Rao et al. [31], with adaptations. For this determination, three samples of 200 mg of fresh material were collected from each incubation time (60 and 120 min) and the control treatment. For the extraction procedure, samples were macerated and then homogenized with an extraction buffer composed of 50 mM potassium phosphate buffer (pH 7.0) (1/5; *w*/*v*), followed by centrifugation at 12,000 rpm for 20 min at 4 °C. The supernatant was collected and used as a culture substrate in the reaction mixture for the determination of the activity of each enzyme.

The SOD (EC 1.15.1.1) activity was determined using 80 μL of culture substrate mixed with 2 mL of 50 mM potassium phosphate buffer (pH 7.8) containing 10 mM methionine, 56 μM nitroblue tetrazolium, and 24 μL of 100 mM riboflavin. The reaction mixture was made in duplicate samples, in which one sample was maintained under dark conditions to be used as a blank and the other was illuminated for 15 min in a box line with aluminum foil (using a 15 W white lamp at a distance of approximately 12 cm from samples). The SOD activity was determined by the reading at 560 nm using a standard curve, which was constructed using purified SOD as the standard.

The CAT (EC 1.11.1.6) activity was determined using 20 μL of culture extract mixed with 180 μL of 50 mM potassium phosphate buffer (pH 7.0) containing 13.3 mM hydrogen peroxide. The CAT activity was determined at 240 nm by the absorbance decreases for 3 min at 25 °C, using the molar extinction coefficient of hydrogen peroxide (39.4 mM cm^−1^).

The APX (EC 1.11.1.11) activity was determined using 40 μL of culture extract mixed with 160 μL of potassium phosphate buffer (pH 7.0) containing 1.5 mM ascorbic acid and 2 mM hydrogen peroxide. The APX activity was determined at 290 nm by the absorbance decreases for 2 min at 25 °C, using the molar extinction coefficient of ascorbic acid (2.8 mM cm^−1^).

The GPX (EC 1.11.1.7) activity was determined using 7 μL of culture extract (diluted in extraction buffer 1:80, *v*/*v*) mixed with 193 μL of 10 mM sodium phosphate buffer (pH 6.0) containing 0.125% (*v*/*v*) H_2_O_2_ and 0.250% (*v*/*v*) guaiacol. The GPX activity was determined at 470 nm by the absorbance decreases for 3 min at 30 °C, using the molar extinction coefficient of guaiacol (25.2 mM cm^−1^).

Protein contents were quantified by the Bradford method [32] at 595 nm, using bovine serum albumin (BSA) as a standard. The activities of SOD, CAT, APX, and GPX were expressed in μKatal. mg^−1^ of protein (1 katal signifies the amount of enzyme that catalyzes 1 μmol of a substrate in 1 min).

### 3.7. Fluorescein Diacetate (FDA) Analysis

FDA staining was used to monitor cell viability during and following the cryopreservation procedure. A stock solution of 1% (weight: volume–FDA: acetone) was prepared and to 2% (volume: volume) in water. Approximately 10 μL of the diluted solution was added directly to approximately 30 mg of EC per 5 min. This was followed by the observation of green fluorescence under UV illumination using an inverted microscope (Olympus IX81) coupled with a digital camera (Olympus DP71, Shinjuku, Tokyo, Japan) and the software CellSens Dimension (Olympus). Three representatives and independent samples were analyzed in the following cryopreservation steps: (i) after incubation in cryoprotection solution; (ii) after freezing and thawing; (iii) 60 days after regrowth.

### 3.8. Statistical Analysis

All the statistical analyses were performed using GraphPad Prisma Version 8.1.1. Data obtained for the increased ratio in phytotoxicity test, regrowth rates, and the quantification of MDA, H_2_O_2_, SOD, CAT, APX, and GPX were subjected to a one-way analysis of variance (ANOVA), followed by Tukey mean separation test (*p* < 0.05). Linear regression was used to evaluate the results of the phytotoxicity test. A correlation analysis was used to estimate the interactions between the data obtained for the quantification of MDA, H_2_O_2_, SOD, CAT, APX, and GPX.

## 4. Discussion

### 4.1. Cryopreservation Procedure

For several plant species, embryogenic cultures can be efficiently conserved for a long time without the loss of embryogenic potential through the cryopreservation technique. However, there are no reports on the cryopreservation of the somatic embryos of bamboo species, which may be a consequence of the complexity of somatic embryogenesis, the few somatic embryogenesis protocols established, and the scarce information available about this group of plants [11]. Based on this, the need for studies to establish strategies for the in vitro conservation of bamboo is evident, especially based on cryopreservation approaches.

Cryopreservation is a complex process wherein the achievement of high survival rates depends on the optimization of many factors, including the correct choice of explant. The standardization of the culture’s conditions and the use of appropriate intervals between subcultures can be determinant factors for the success of cryopreservation [33]. It is suggested that cultures in the exponential phase, in active growth, and which exhibit cells with a low water content, least volume, small vacuoles, and dense cytoplasm, resulting in higher cytosol: vacuole ratio and thus a greater solute concentration, present an improved tolerance to the freezing process [5,18,34]. Our growth dynamics results revealed that the embryogenic cultures of *G. chacoensis* are in the exponential/log phase for days 35 to 45 of culture, suggesting that this is the best period for the cryopreservation protocol. However, this behavior should be observed case by case, since each species presents a different growth curve, reinforcing the importance of the analysis of cultures’ growth dynamics before cryopreservation.

Different methodologies can be applied for EC cryopreservation, including two-step, slow-freezing, vitrification, and encapsulation/dehydration [2]. The choice of the technique depends on the material type and characteristics. Among these methodologies, the most recommended for the cryopreservation of an embryogenic callus suspension culture is the two-step, slow-freezing method, which has been widely reported for species of conifers [7,8,35], bananas [36], and herbaceous and hardwood species [5]. No works have been reported for bamboos species, emphasizing the importance of researching cryopreservation strategies for this group of plants. The purpose of the use of the two-step, slow freezing protocol is to: a) expose the cells to a high-concentration solution to stimulate dehydration and b) to use appropriate cooling conditions and cryoprotective agents in such a manner that the intracellular content becomes viscous and forms the so-called “glassy state”, avoiding the formation of harmful crystal ice [37].

Therefore, the present study established a two-step, slow-freezing cryopreservation protocol for the suspension of cultures of *G. chacoensis* ECs using preincubation in a culture medium with an osmotic agent (mannitol 180 g L^−1^) for two days, followed by incubation in a cryoprotective solution with four kinds of cryoprotective agents: sucrose (2 M), glycerol (1 M), DMSO (1 M; 7.1%), and proline (1%), and slow-cooling using Mr. Frosty per 4 h before immediate immersion in LN.

### 4.2. Cryoprotective Agents and Their Importance for Increasing Cell Tolerance to Dehydration and Freezing Steps

The choice of cryoprotective, the concentration, and the time of incubation used are germplasm-dependent and should be optimized to avoid possible toxic effects that can affect cell viability. Cryoprotective agents (CPAs) facilitate cell cytosol concentration, avoid ice crystal formation, and increase cell tolerance to dehydration and freezing [17]. Indeed, the purpose of using these CPAs is to properly combine them with the optimal cryopreservation methodology so that ice formation is negligible [17]. Two types of cryoprotective agents can be used in cryopreservation procedures: ones which are permeable (penetrating) for the membrane, including DMSO, glycerol, and non-ionic molecules, and ones which are not permeable (non-penetrating) for the membrane, including polyethylene glycol and sucrose [37]. It is suggested that the combination of more than one agent in a lower concentration, as was used in our work, appears to be more appropriate than the use of only one agent in a high concentration for achieving higher viability and cell survival [5]. Thus, optimizing the CPA concentration and time of exposure is crucial, as toxicity and permeability seem to be germplasm-dependent [17].

Our phytotoxicity test results revealed that the incubation of *G. chacoensis* ECs for 30 and 60 min in a cryoprotective solution did not affect their growth dynamics. However, incubation for 120 min was deleterious after the twentieth day in culture, and incubation for 240 min was phytotoxic. Inversely, for the *Araucaria angustifolia* embryogenic cell lines, no inhibitory effects were observed with the same cryoprotective solution used in our study, even with an incubation period of 240 min [7]. Indeed, the time of exposure varies among species, and optimization is necessary for the success of the cryopreservation procedure. Salaj et al. [35] reported a 60 min incubation for embryogenic cultures of *Pinus Nigra* using a cryoprotective solution with 18% sucrose and 7.5% DMSO, followed by slow-freezing, while Lambardi et al. [5] proposed that 30 min of incubation presented the highest callus regrowth when compared to 60 and 90 min of incubation in the two-step freezing cryopreservation of Cypress embryogenic masses using a solution containing 180 g·L^−1^ of sucrose and 7.5% DMSO; they also reported that conversion rates were not affected when compared to the control. Based on our phytotoxicity test, we used 60 and 120 min incubation times in our further cryopreservation procedure.

### 4.3. Regrowth Rates and Cell Viability after Cryopreservation

The analysis of regrowth after cryopreservation revealed a 100% cell survival rate, independent of the time of incubation (60 or 120 min), and the cultures presented a 10% increase ratio in fresh material at 60 days after cryopreservation. In fact, increased fresh material may be a good indicator of cell re-establishment after cryopreservation [34]. In addition, the results of our FDA analysis showed that, although *G. chacoensis* ECs may suffer some stress during the incubation in the cryoprotective solution, the viability of the cells was maintained. Additionally, the highest viability was observed 60 days after regrowth, suggesting that our protocol allowed for the successful cryopreservation of ECs of *G. chacoensis*. The FDA test is commonly used to verify cell viability during cryopreservation [7,18,34,35]. In this assay, cell wall esterase cleaves fluorescein diacetate and fluoresce green. In this way, only cells with intact plasma membranes are able to fluoresce, while non-viable cells appear colorless [38].

Previous studies have shown that the regrowth and conversion rates after cryopreservation were directly affected by the time of exposure to cryoprotectant treatment and the kind of cryoprotectant used. Delgado-Aceves et al. [39] revealed that the regrowth and conversion of *Agave* somatic embryos achieved higher rates after exposure for 15 and 30 min in a plant vitrification solution (composed of glycerol, DMSO, ethylene glycol, and sucrose) when compared to exposures of 45 and 60 min, suggesting that lower incubation times in pretreatment solution may result in major recovery and conversion rates. Salaj et al. [40] indicated that the regrowth rate after cryopreservation of ECs of different cell lines of *P. nigra* were affected by the pre-treatment used, in which sorbitol and maltose pre-treatment resulted in less regrowth than sucrose and cryopreservation did not affect the conversion and proliferation of cultures. In our study, we established a successful cryopreservation protocol with 100% survival rates using both 60 and 120 min incubation. However, there is still a gap in the maturation and conversion phases of *G. chacoensis* ECs, showing that further studies should be applied to optimize these steps.

One of the possible reasons for the success of our cryopreservation procedure is the correct establishment of important critical factors, such as the choice of the explant growth period, the use of an efficient pre-treatment, a suitable combination of cryoprotective agents, a suitable time of exposure, an efficient thawing procedure, and the selection of the cryopreservation methodology (slow-cooling using Mr. Frosty). Moreover, our study represents important progress in the cryopreservation of bamboo, being the first work to describe a complete protocol for the cryopreservation of embryogenic cultures of a bamboo species.

### 4.4. CAT and APX as Central Antioxidant Enzymes in Maintaining G. chacoensis Embryogenic Cultures’ Cell Redox Homeostasis, Assuring Successful Cryopreservation

Cryopreservation is a process that imposes significant stress on cells. Monitoring the activity of antioxidant enzymes during cryopreservation may provide a better comprehension of plant metabolism and response to the production of ROSs [21,22]. The analysis of the redox state realized in the present study revealed that the incubation of *G. chacoensis* ECs in the cryoprotectant solution resulted in a considerable increase in MDA content, independent of the time of incubation used—60 or 120 min—in addition to a high H_2_O_2_ content and CAT activity. Interestingly our correlation analysis showed a strong positive correlation between MDA and H_2_O_2_ and MDA and CAT, proposing a relationship between the data. 

Our result of an increased MDA content following incubation in the cryoprotective solution may suggest the occurrence of oxidative stress, implying that this is a critical step in the cryopreservation of *G. chacoensis* ECs. As a result of oxidative stress, plant cells may have two different responses: (1) the activation of the antioxidant enzymatic defense system and (2) an increase in lipid peroxidation; for cells survival after cryopreservation, the first reaction is positive and the second is negative [9]. In fact, previous studies have associated an increase in MDA content—the product of lipid peroxidation—with oxidative stress and the generation of ROSs during different steps of cryopreservation [9,41,42,43]. Additionally, excessive MDA production is recognized as one of the main factors responsible for low viability after cryopreservation [9,23,24,25].

Enhanced levels of H_2_O_2_ found in our work may be associated with oxidative stress as well as with the low activity of SOD, since SOD is the primary enzyme in the H_2_O_2_ scavenging [44]. Low SOD activity and high MDA content can negatively affect the survival of cultures after cryopreservation, leading to a higher occurrence of cell ultrastructure damages, i.e., plasmolysis, rupture of the nuclear envelope, and condensation of mitochondria [42]. Moreover, Ren et al. [45] suggested that H_2_O_2_ was the principal ROS molecule involved with oxidative stress during the cryopreservation of *Arabidopsis* seedlings, but an enhancement in CAT activity could efficiently scavenge them. Nevertheless, it seems that the ECs of *G. chacoensis* may respond to oxidative stress by the activation of CAT, the antioxidant enzyme which presents the highest turnover rate for H_2_O_2_ dismutation (6 × 10^6^ molecules per min) [44].

In the following step evaluated, after thawing, our results demonstrated a decrease of 40% and 50% in the MDA and H_2_O_2_ content, respectively, together with an increase in CAT and APX activities. In addition to this, correlation analysis revealed a positive correlation between MDA and H_2_O_2_ and CAT and APX and an inverse negative correlation between H_2_O_2_ and CAT, proposing that CAT and APX may be acting as efficient ROS scavengers, resulting in the reduction of oxidative stress and, consequently, decreases in the content of H_2_O_2_ and MDA, even after freezing and rewarming steps, which were normally critical steps in cryopreservation procedures.

The importance of the activation of antioxidant enzymes for ROS scavenging during cryopreservation is well known. Viana et al. [41] have shown that an enhancement in CAT and APX after osmoprotective treatment in the cryopreservation of shoot tips of *Passiflora suberosa* was decisive for H_2_O_2_ scavenging. Chen et al. [46] proposed that an increase in CAT activity seems to be related to cryogenic stress tolerance and results in increased recovery rates for cryopreserved *Arabidopsis* seedlings. James Antony et al. [47] suggested that the low regeneration rates obtained for cryopreservation by vitrification of the orchid *Dendrobium* seem to be associated with the low activity of CAT, POX, and APX during critical steps of the protocol. Poobathy et al. [48] also proposed that low CAT activity after the post-thawing stages resulted in low survival rates of cryopreserved, protocorm-like bodies of *Dendrobium.* An increased activity of APX and CAT during the dehydration and cryostorage treatment steps in the cryopreservation of the orchid *Brassidium* indicated the occurrence of high oxidative stress levels [49]. Indeed, these studies support the hypothesis that the activation of the antioxidant enzymes CAT and APX may be crucial for safeguarding cellular homeostasis in response to the oxidative stress generated after the incubation of G. *chacoensis* ECs in the cryoprotective solution. In the same way, the increased activity of antioxidant enzymes also assures enhanced cold tolerance for the cultures, as these enzymes function as efficient ROS scavengers that protect the cell from the excessive ROSs produced in low-temperature conditions, as mentioned by Baek and Skinner [50].

In the regrowth phase, no significant difference was observed for either antioxidant enzyme activity or the MDA and H_2_O_2_ content between the cryopreserved materials and the control, suggesting that although oxidative stress was observed in the initial steps of cryopreservation protocol, a complete recovery of the cryopreserved ECs of *G. chacoensis* was achieved 60 days after regrowth. Therefore, the activation of the antioxidant enzymes CAT and APX seems to be crucial in safeguarding cell redox homeostasis and assures the maintenance of cell viability after cryopreservation.

## 5. Conclusions

In this paper, we described for the first time the successful cryopreservation for *G. chacoensis* ECs using a two-step slow freezing protocol. Our results suggested that both incubation times in the cryoprotective solution—60 and 120 min—were effective and led to similar regrowth rates and fresh material increase ratios. Regarding the ECs’ redox state after cryopreservation, our results suggested that incubation in the cryoprotective solution was the most critical step, having observed an increase in oxidative stress levels. However, the rapid activation of the antioxidant defense system, mainly through the enhancement of the CAT and APX activities, in this same step as well as after thawing, resulted in the re-establishment of cell redox homeostasis, suggesting that these enzymes may function as cryoprotectants that assure cell viability maintenance and regrowth after cryopreservation.

## Figures and Tables

**Table 1:** Correlation values obtained for MDA, H_2_O_2_, SOD, CAT, APX, and GPX of *G. chacoensis* embryogenic cultures in each evaluation time: after cryoprotective solution, after thawing, and after regrowth.

		MDA	H_2_O_2_	SOD	CAT	APX	GPX
**Cryoprotective**	**MDA**	1					
	**H_2_O_2_**	0.788 *	1				
	**SOD**	−0.729 *	−0.414	1			
	**CAT**	0.906 **	0.584	−0.614	1		
	**APX**	−0.304	−0.155	0.628	−0.333	1	
	**GPX**	−0.576	−0.439	0.910 **	−0.367	0.704 *	1
**Thawing**	**MDA**	1					
	**H_2_O_2_**	−0.797 **	1				
	**SOD**	0.294	−0.083	1			
	**CAT**	0.658	−0.770 *	0.393	1		
	**APX**	0.724 *	−0.590	0.753 *	0.821 **	1	
	**GPX**	0.286	−0.419	0.697 *	0.704 *	0.699 *	1
**Regrowth**	**MDA**	1					
	**H_2_O_2_**	0.253	1				
	**SOD**	0.416	0.359	1			
	**CAT**	0.382	−0.247	0.158	1		
	**APX**	0.437	0.670 *	0.110	−0.357	1	
	**GPX**	0.382	−0.549	−0.213	0.307	0.092	1

* and ** indicate significant differences at *p* < 0.05 and *p* < 0.01, respectively.

## Data Availability

The data presented in this study are available on request from the corresponding author. The data are not publicly available due to further studies in development.

## Supplementary Materials

The following supporting information can be downloaded at: https://www.mdpi.com/article/10.3390/plants12030673/s1.
