# Supplementary material for: Enzymatic Antioxidant System Activation Assures the Viability of Guadua chacoensis (Bambusoideae, Poaceae) Embryogenic Cultures during Cryopreservation
Source: Plants (Basel). 2023 Feb 3;12(3):673. doi: 10.3390/plants12030673 (PMC9920021; doi:10.3390/plants12030673)
Supplement: Supplementary file 1 [file plants-12-00673-s001.zip › plants-2142722-supplementary.pdf]

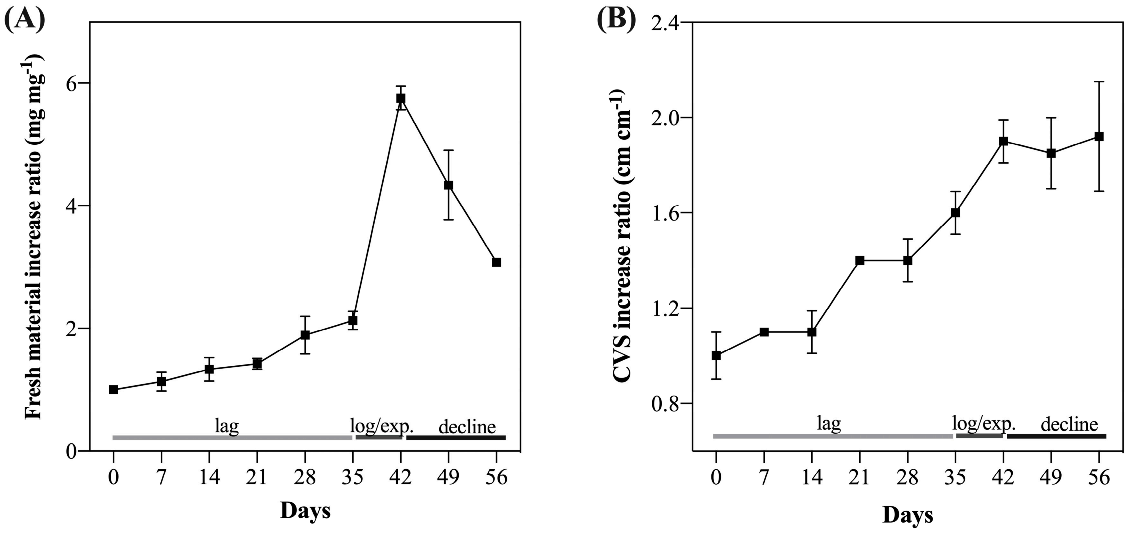

Supplementary Figure S1 Increase ratio of growth dynamics of *G. chacoensis* embryogenic cultures measured by a destructive method **(A)** and non-destructive method **(B)**. **(A)** increase ratio based on fresh weight (mg mg^{-1}); **(B)** increase ratio based on CVS (cm cm^{-1}). Bars indicate the standard deviation of the mean. Lag: lag phase; log/exp: logarithmic or exponential phase; decline: decline phase.
